# Effect of Rhizobacteria Inoculation via Soil and Seeds on *Glycine max* L. Plants Grown on Soils with Different Cropping History

**DOI:** 10.3390/microorganisms10040691

**Published:** 2022-03-23

**Authors:** Denise Almeida Fonseca Fiuza, Luciana Cristina Vitorino, Edson Luiz Souchie, Moacir Ribeiro Neto, Layara Alexandre Bessa, Cintia Faria da Silva, Natasha Taline Trombela

**Affiliations:** 1 Laboratory of Agricultural Microbiology, Instituto Federal Goiano, Campus Rio Verde, Highway Sul Goiana, Km 01, Rio Verde 75901-970, GO, Brazil; denisefiuza@hotmail.com (D.A.F.F.); edson.souchie@ifgoiano.edu.br (E.L.S.); moacir@agropotencia.com.br (M.R.N.); cintiafsbio@hotmail.com (C.F.d.S.); natashatrombela18@gmail.com (N.T.T.); 2 Laboratory of Metabolism and Genetics of Biodiversity, Instituto Federal Goiano, Campus Rio Verde, Rio Verde 75901-970, GO, Brazil; layara.bessa@ifgoiano.edu.br; 3 Laboratory of Plant Mineral Nutrition and CEAGRE, Exponential Agriculture Center of Excellence, Instituto Federal Goiano, Campus Rio Verde, Rio Verde 75901-970, GO, Brazil

**Keywords:** plant-growth-promoting bacteria, bioinoculants, inoculation methods, microbial biomass

## Abstract

Field experiments testing the effect of phosphate-solubilizing rhizobacteria (PSRB) should consider the cropping history and the method used to inoculate the strains. We evaluated the hypothesis that PSRB previously isolated from soybean seedlings could be effective in promoting growth in this oilseed crop in soils with different cultivation periods. We also evaluated whether this growth promotion could be influenced by cultivation histories or the inoculation method (via seeds or soil). Thus, we conducted an experiment in five fields cultivating *Glycine max* during two seasons (2019/2020 and 2020/2021), to test the effectiveness of PSRB (SAF9-*Brevibacillus* sp., SAF11-*Brevibacillus* sp., and SAC36-*Bacillus velezensis*) compared with results observed for the inoculant BiomaPhos (mix of *Bacillus subtilis* and *Bacillus megaterium*). The present study was based on the evaluation of vegetative growth, nutritional and yield parameters, and microbial biomass carbon (MBC). PSRB were more effective than, or showed similar effectiveness to, BiomaPhos for most of the evaluated vegetative, nutritional, and yield characteristics. In the fields tested in the summer 2019/2020 crop, SAC36 and SAF9 strains stood out as growth promoters, whereas in the 2020/2021 crop, SAF11, SAC36, and BiomaPhos were notable. There did not seem to be a direct relationship between long histories of soybean cultivation as a monoculture and low yield in the field. However, yield seems to be associated with soil nutritional characters such as Ca, Mg, K, P, cation exchange capacity, and organic matter levels. PSRB inoculation positively affected nodulation (NN) and nodule dry mass (NDM) in the evaluated fields in the 2019/2020 crop, and the aerial part dry mass (APDM), NN, NDM, yield, and MBC of the evaluated fields in the 2020/2021 crop. In contrast, the inoculation method was observed to have a strong effect on APDM, NN, root dry mass, and MBC, as the plants inoculated via seed showed higher mean values than those in the plants inoculated via soil. This study demonstrated the growth-promoting potential of new phosphate-solubilizing strains, which may eventually be incorporated by the biostimulants market to freely compete with BiomaPhos.

## 1. Introduction

The Cerrado is an important Brazilian grain- and fiber-producing region, which has seen a large increase in cultivable area during the last 30–40 years [1]. Among these crops, soybean (*Glycine max* L.) stands out due to developments in technologies and soil management practices [2]. In the 2020/2021 crop year, the country will become the world’s largest producer of this oilseed, with a production of 136 million tons of grains [3].

Phosphorus is the nutrient that most limits the productivity of agricultural soils in the Cerrado Domain in Brazil. High investments in fertilization are required to ensure adequate P levels, as this is the most expensive nutrient in the fertilization of soybean crops [4]. Although P is abundant in many types of soil, most of it is not readily accessible to plants due to phosphate anions’ high affinity to Fe, Al, and Ca oxides, forming poorly soluble compounds [5]. Therefore, different strategies have been developed to improve P supply to crops, and the most promising technique is the use of rhizobacteria that participate in the transformations of soil P [6,7,8]. At present, in Brazil, the inoculant BiomaPhos, developed to promote plant growth through the action of phosphate-solubilizing bacterial strains, is the only available product on the market. The introduction of other solubilizing strains in the biostimulants market is urgently required, as such bacterial activity is always associated with improved efficiency in P use and increased productivity [9,10,11,12].

Plant-growth-promoting rhizobacteria have been associated with several bacterial genera, including *Azospirillum*, *Pseudomonas*, *Enterobacter*, *Bacillus*, *Paenibacillus*, *Serratia*, *Burkholderia*, *Brevibacillus*, and *Klebsiella* [13,14,15,16]. Many studies report on the positive effects of rhizobacteria inoculation in increasing tissue nutrient concentration and improving the growth and productivity of *G. max*, either under laboratory conditions or greenhouse trials [14,17,18,19]. However, in the field, the use of rhizobacteria has been hampered by results that are often inconsistent with those observed at the small scale [20,21,22]. Understanding of new strains of rhizobacteria and their readiness for the agricultural market is affected by factors such as lack of persistence in the soil, competitiveness between the introduced soil microorganisms and the resident microbial community, and scarce knowledge of the numerous plant–microorganism interactions [23,24,25].

Experiments conducted in the field should consider potential adverse factors, such as the history of the cropping field and the initial nutritional status of the soil, because soil quality is profoundly dependent on management practices [26,27]. This is especially true in fields that have been subjected to years of consecutive cultivation in monoculture systems, as was the case in most of the fields evaluated in this study. Karlen et al. [28] define soil quality as a soil’s capacity to function in an ecosystem to support plants and animals, resist erosion, and reduce negative impacts on associated water and air resources. From this perspective, crop sustainability is directly associated with factors such as the availability of organic matter and essential nutrients, such as N, P, and K, in the soil [29,30,31]. However, microorganisms used in the formulation of inoculants can contribute to the enrichment of the edaphic microbial community and increase the microbial biomass, which is an important index of soil quality [32,33], and are commonly associated with crop productivity and sustainability [34].

Inocula are differentially resistant to abiotic and biotic stresses when made available to soil, and seed inoculation techniques used for research purposes are often not feasible on a commercial scale. Furthermore, maintaining inoculum viability throughout commercial seed treatment and storage processes faces significant technical challenges [35]. Thus, the choice of inoculation method seems to be a critical step when aiming for good yields in soybean crops, because the colonization rate may depend on how the plant is exposed to the inoculum [36,37].

Considering all these factors, we conducted an experiment in five fields of *G. max* cultivation to evaluate the hypothesis that phosphate-solubilizing rhizobacteria, previously isolated from soybean seedlings, could effectively promote the growth of this species in soils with different soybean cropping histories. This study compared its results with those observed for the commercial product BiomaPhos to test the effectiveness of these rhizobacteria. We also investigated the hypothesis that the cultivation history of the planted fields can alter the effect of plant-growth-promoting rhizobacteria on soybean cultivation, and that inoculation via seeds is superior to inoculation via soil.

## 2. Materials and Methods

### 2.1. Multifunctional Rhizobacteria Collection and Inoculum Preparation

Multifunctional rhizobacteria were previously isolated from *G. max* seedlings grown on eutrophic red Latosol, sampled from an agricultural field with a 30-year history of soybean cultivation, located in the municipality of Indiara, in the southwest of the state of Goiás, Brazil. Three rhizobacteria were chosen from 139 strains that were previously isolated from *G. max* seedlings and tested in vitro for their multifunctional potential. These three rhizobacteria stood out when evaluated for efficiency in the solubilization of CaHPO_4_ and FePO_4_ (mg L^−1^ in GL culture medium), synthesis of the phytohormones indole acetic acid (IAA) and gibberellin (µg mL^−1^), production of siderophores, and antibiosis to the phytopathogenic fungi *Fusarium* sp. and *Sclerotinia sclerotiorum* (R.I.%, percentage of relative inhibition of mycelial growth in the phytopathogen in paired cultures) (data undergoing publication process) (Table 1).

The isolated rhizobacteria were refrigerated at 12 °C and separately reactivated in nutrient agar culture medium (meat extract, 3 g; peptone, 5 g; and agar, 25 g L^−1^) at 28 °C for 48 h. The inocula were then prepared in 2 L nutrient broth medium (meat extract, 3 g; peptone, 5 g). The cell concentration of each suspension was standardized with OD_600_ (optical density at 600 nm) of 0.8, according to the correlation between optical density and the number of colony-forming units (CFU mL^−1^). The adjustment was made using saline solution, and the OD_600_ of 0.8 corresponded to approximately 10^9^ CFU mL^−1^ of the suspensions. The inoculum suspensions were refrigerated until use.

### 2.2. Preparation, Installation, and Conduction of Field Experiments

#### 2.2.1. Characterization of the Experimental Fields

**Figure 1:**
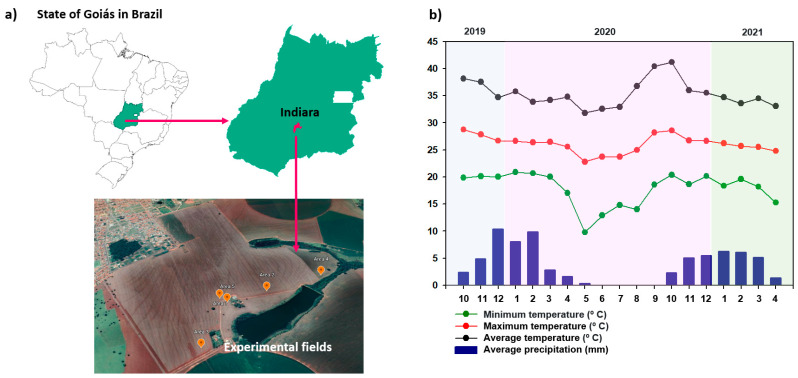
Location of five experimental fields in the municipality of Indiara, interior of the state of Goiás, Brazil (**a**) and observed data for minimum, average, and maximum temperatures and average rainfall during the summer crops 2019/2020 and 2020/2021 (**b**). Data source: agritempo.gov.br (accessed 20 January 2022).

All the experimental fields were located in the municipality of Indiara, interior of the state of Goiás, Brazil (Figure 1a). Five commercial soybean production fields were used consecutively, with the following coordinates: Field 1 (17°09′27.82″ S, 50°00′29.38″ W; altitude of 589 m), Field 2 (17°09′41.70″ S, 50°00′29.18″ W; altitude of 587 m), Field 3 (17°09′20.68″ S, 50°00′45.88″ W; altitude of 596 m), Field 4 (417°100.02″ S, 50°00′25. 50″ W; altitude of 577 m), and Field 5 (17°09′29.63″ S, 50°00′31.36″ W; altitude of 585 m). The regional climate, according to the Köppen classification, is Aw, characterized as tropical, with hot summers and a tendency towards high levels of precipitation, and dry winters, with a dry season between May and September.

The total rainfall during the first period of experiments, from 1 October 2019 to 30 April 2020, was 1215.5 mm, with average minimum and maximum temperatures of 19.8 °C and 35.7 °C, respectively. For the second period, from 1 October 2020 to 30 April 2021, the rainfall was 957.6 mm, with average minimum and maximum temperatures of 18.6 °C and 35.5 °C, respectively (Figure 1b).

The experimental fields were chosen according to their cultivation history: Field 1, soybean production for 30 years; Field 2, soybean production for 15 years; Field 3, soybean production for 10 years; Field 4, second year of soybean cultivation; and Field 5, first year of soybean cultivation.

The experimental plots were 8.0 m long × 3.0 m wide (six rows spaced 0.50 m), and the total area of each plot was 480 m^2^. The tests were conducted during two consecutive crops, 2019/2020 and 2020/2021, and all necessary and recommended phytosanitary treatments for soybean cultivation in the region were followed.

Before the installation of the experiments, three soil samples were collected from each field for chemical and granulometric characterization. The sampling was always performed in the 0–20 cm depth layer, and the soil was classified as eutrophic Red Latosol [38].

#### 2.2.2. Experimental Procedures in the Summer Crops of 2019/2020 and 2020/2021

In the 2019/2020 summer crop, the experiments were conducted in Fields 1, 2, and 5. After sowing, each rhizobacteria inoculum was applied to the soil at a concentration of 10^9^ CFU mL^−1^ using a backpack pump at a flow rate of 150 mL ha^−1^ (0.36 mL per plot). Seeds of the cultivar M7110 IPRO were used at a density of 40 seeds m^2^. The seeds were previously inoculated with *Bradyrhizobium* spp. and *Azospirillum brasilense* AbV5 and AbV6 (2 × 10^8^ CFU mL^−1^), and treated with fungicide and insecticide. In this treatment, we used the insecticides Cruiser 350 FS (Tiametoxam 350 g L^−1^), at the recommended dose of 200 mL per 100 kg of seeds, and Avicta 500 FS (Abamectin 350 g L^−1^), at a dose of 100 mL per 100 kg of seeds. The product Maxin Advanced (Metalaxyl-M 20 g L^−1^, Fludioxonil 25 g L^−1^, and Thiabendazol 150 g L^−1^) was used as a fungicide at a dose of 100 mL per 100 kg of seeds. Sowing occurred on 6 December 2019, with fertilizer (at 300 kg ha^−1^ of NPK, produced at concentrations of 6 kg of N, 40 kg of P, and 10 kg of K for every 100 kg of fertilizer) applied directly in the planting furrow. Plants grown in soil without inoculum, and in soil treated with the commercial product BiomaPhos (mix of *Bacillus subtilis* CNPMS B2084-BRM034840 and *Bacillus megaterium* CNPMS B119-BRM033112), were used as control treatments.

The experiments in Fields 3 and 4 were conducted during the 2020/2021 summer crop. In these tests, the rhizobacterial inocula were directly incorporated onto the soybean seeds. The seeds were initially treated with fungicide and insecticide, as mentioned above, at the same doses. The number of seeds required for each plot was calculated (192 g), and the seeds were placed in polyethylene bags and separately treated with 0.36 mL of each inoculum at a concentration of 10^9^ CFU mL^−1^, diluted in 20 mL of sterile water. The material was agitated for 60 s to facilitate adherence of the inoculum to the seeds. The treated seeds were arranged on a layer of aluminum foil, in the shade, and were immediately planted when dry. Sowing was performed on 13 November 2020 at a density of 20 seeds linear m^−1^ with fertilizer (at 380 kg ha^−1^ of PK, produced at concentrations of 20 kg of P and 20 kg of K for every 100 kg of fertilizer) directly applied to the planting furrow. Plants from non-inoculated seeds and from seeds treated with BiomaPhos were used as control treatments.

### 2.3. Data Collection

For each inoculation treatment, five soybean plants were randomly sampled per plot at the R2 reproductive stage and the following vegetative growth parameters were determined: plant height (cm), aerial part and root dry mass (APDM and RDM-g), number of nodules (NN), nodules’ dry mass (NDM-mg), total N and P levels in the aerial part and grains (g kg^−1^), mass of one thousand grains (g), and productivity (kg ha^−1^). Aerial height was evaluated by measuring the distance from the neck of the plant to the end of the canopy. The aerial part and roots were separated, conditioned in paper bags, placed to dry in an oven with forced air circulation under constant temperature (65 °C) for 72 h, and then weighed to determine their dry mass. Samples of the aerial part were ground in a Willey mill and stored in plastic bags for determination of the N and P levels, according to Malavolta [39].

To evaluate root nodulation, plants were collected using a cutting spade, centering the plant in the 0.4 (L_1_) × 0.4 (L_2_) m frame, while taking care to maintain the pre-established soil volume for each plant at approximately 0.032 m^3^, with dimensions of 0.4 (L_1_) × 0.4 (L_2_) × 0.2 (H). The roots, nodules, and soil were separated using a 3-mm mesh sieve and running water. The nodules were placed separately in paper bags, identified, and dried in an oven at 65 °C until constant weight. The dry masses were obtained and the NN per plant was counted.

Plants were harvested after physiological maturity (R8) by uprooting them from each plot. They were threshed in a mechanical thresher and the grains were weighed to determine the productivity at 13% humidity (wet basis). A sample from each plot was separated for determination of the mass of 1000 grains on precision electronic scales (0.01 g), and for determination of the total P and N levels in the grains.

At the end of the experiments, the microbial biomass carbon (*MBC*) was measured in soil samples from the different fields. These samples were collected at a depth of 10 cm, and the *MBC* was evaluated according to Silva et al. [40], using the fumigation-extraction method, which consists of extracting the *MBC* after applying chloroform to the samples, causing the death of the microorganisms and release of the cellular components. The calculation of the *MBC* content was performed using the following expression:

$$\frac{MBC=(F-NF)}{Kc}$$

where *MBC* is the *C* of the microbial biomass (mg kg^−1^); *F*, the *C* of the fumigated sample (mg g^−1^); *NF*, the *C* of the non-fumigated sample (mg kg^−1^); and *Kc* the factor used to convert the extracted *C* into *MBC*. This study adopted the *Kc* value of 0.33 suggested by Silva et al. [40], based on climate and type of soil.

### 2.4. Experimental Design and Statistical Analyses

The relationships between the soil characteristics sampled in the different study fields were jointly analyzed with a correlation matrix and combined in a principal component analysis (PCA). As these variables had different measurement units, a correlation PCA was performed and constructed using data standardized to have a mean value of 0 and standard deviation of 1. The number of components was chosen according to the eigenvalues (>1.0) and explained variance (above 80%).

The study evaluated five treatments: inoculation with the strains SAF9 (*Brevibacillus* sp.), SAF11 (*Brevibacillus* sp.), and SAC36 (*Bacillus velezensis*), control without inoculation, and inoculation with the commercial product BiomaPhos. These treatments were evaluated in three fields in the 2019/2020 crop and two fields in the 2020/2021 crop. The experiments were conducted in a randomized block design with four replicates, with each replicate being considered a plot. The effect of the bacteria within each experimental field was evaluated for each individual strain within each crop, as was the effect of the cultivation history. We also evaluated the effect of the inoculation method (comparing different crops) for each tested strain.

The different vegetative growth variables, (nodulation, MBC, and N and P levels of the aerial part and grain, and productivity) were analyzed using analysis of variance, and in cases of differences between the mean values of the treatments, these were compared using the Tukey test at a probability level of 5%. All statistical tests were performed in R software, version 4.0.4 [41].

## 3. Results

**Figure 2:**
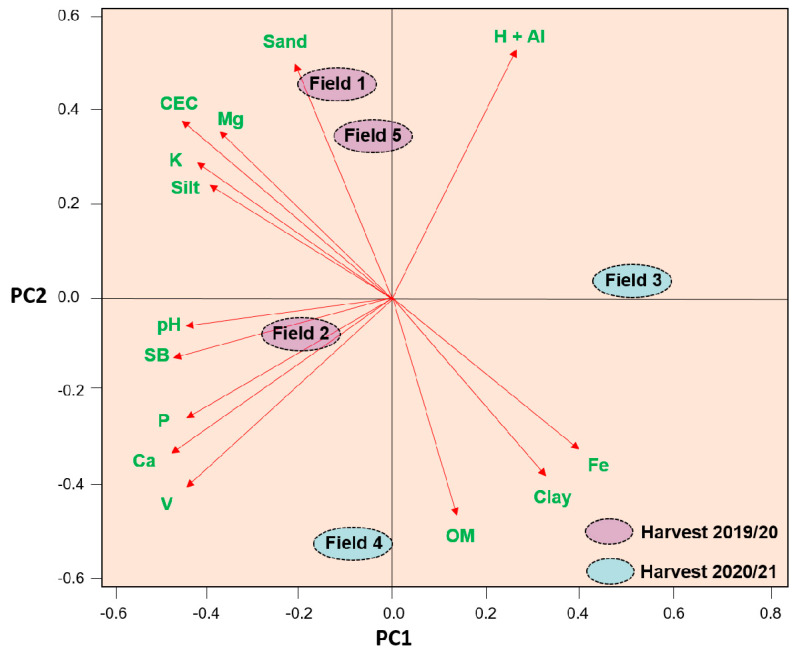
Principal component analysis of the relationship among soil attributes of five experimental fields in the interior of the state of Goiás, Brazil, evaluated for the cultivation of *Glycine max* L. in summer crops of 2019/2020 and 2020/2021. These fields had different histories of soybean cultivation. Field 1, soybean production for 30 years; Field 2, soybean production for 15 years; Field 3, soybean production for 10 years; Field 4, second year of soybean cultivation; Field 5, first year of soybean cultivation. OM, organic matter; CEC, cation exchange capacity; SB, sum of bases; V, base saturation (%); and H + Al, potential acidity.

The analyzed fields varied in their nutritional attributes; Fields 1 and 5 in 2019/2020 had high levels of sand (60.73 and 65.75 g kg^−1^, respectively) (Figure 2).

Fields 1 and 2 contained the highest levels of Ca (3.93 and 4.01 cmolc dm^−3^, respectively), Mg (1.17 and 1.38 cmolc dm^−3^, respectively), K (1.10 and 0.87 cmolc dm^−3^, respectively), P (24.8 and 31.30 mg dm^−3^, respectively), and the highest cation exchange capacity (9.63 and 8.58 cmolc kg^−1^, respectively). In contrast, Field 4 contained high levels of organic matter (OM) (37.10 g dm^−3^) and high base saturation (86.07%). Field 3 was the most divergent, with acid soils (pH 4.83), low concentrations of the nutrients Ca, Mg, K, and P (2.03, 0.75, and 0.64 cmolc dm^−3^ and 7.80 mg dm^−3^, respectively) and high concentrations of Fe (59.88 mg dm^−3^) and clay (52.00 g kg^−1^).

### 3.1. Results for the 2019/2020 Summer Crop

The inoculation treatments with the different isolates did not affect plant height in any of the fields evaluated in the 2019/2020 crop year (Table 2), nor did the treatments affect the APDM in Fields 1 and 2; however, in Field 5, the highest mean APDM was observed in plants inoculated with the isolates SAF9 and SAC36, followed by those treated with BiomaPhos and SAF11 (Table 2). Similar patterns were observed for RDM, which was only affected by inoculation treatments in Field 5, with the highest mean observed in plants inoculated with SAC36, followed by those treated with BiomaPhos, SAF9, and SAF11. However, these treatments affected NN and NDM in all three tested fields in the 2019/2020 crop. In Field 1, plants treated with the SAF9 bacterium showed the highest NN, followed by those inoculated with BiomaPhos and SAC36. In Field 2, plants grown with SAC36, SAF11, and BiomaPhos developed more nodules, whereas in Field 5, only plants using the control treatment, not inoculated plants, showed a reduced NN. In Field 1, plants inoculated with BiomaPhos had a high mean NDM, followed by those treated with SAC36 and SAF9, whereas in Field 2, only plants undergoing the control treatment had a low NDM. In Field 5, plants inoculated with SAC36 and BiomaPhos exhibited the highest mean NDM, followed by those treated with SAF9. In general, the observed mean NN and mean NDM were always lower in non-inoculated plants than in inoculated plants, regardless of the field.

There was no effect of cultivation history on the measures of plant height, APDM, and RDM when individually comparing the effect of each field tested in the 2019/2020 crop, for each bacterium. However, the mean NN values in Fields 1 and 2 were higher than those in Field 5 in all the tested strains. Similarly, the mean NN values in Fields 1 and 2 were higher than those in Field 5 in the treatments with the bacteria SAF11 and SAC36, whereas Field 1 proved to be superior for SAF9 and BiomaPhos.

The inoculation treatment with plant-growth-promoting rhizobacteria affected the accumulation of N in the aerial part of plants grown in Field 2, with the highest mean values observed in the plants inoculated with SAC36, followed by those treated with SAF9 (Table 3). In contrast, the accumulation of P in the aerial part of the plant was only affected by inoculation treatments in Field 1, where the highest average P was observed in plants inoculated with SAC36 and SAF9. The bacterium SAC36 positively affected the accumulation of N in plants grown in Field 1, whereas, in Field 2, plants treated with SAF9 and BiomaPhos showed the highest accumulation of N in the grains, followed by those treated with SAF11. In Field 5, low concentrations of N in the grains were only observed in the non-inoculated control plants.

The P that accumulated in the grains was only affected by the inoculation treatments in plants grown in Field 2, where the highest mean P was observed in plants treated with SAF9, followed by plants treated with BiomaPhos and SAF11. The accumulation of N in the aerial part only differed among fields with different cultivation histories in the control plants and plants treated with SAC36, and the mean values observed for Fields 1 and 2 were higher than those of Field 5. However, the accumulation of P in the aerial part was only affected by the cultivation history in plants inoculated with SAF9, with higher mean values observed for Field 1, and BiomaPhos, with higher mean values observed in Fields 1 and 2. Regarding the accumulation of N in the grains, plants grown in Fields 1 and 2 showed higher mean values than those of Field 5 in the control treatment and when inoculated with SAF11, but when inoculated with SAF9 and BiomaPhos, plants of Field 2 tended to demonstrate higher N levels in the grains. However, when inoculated with SAC36, plants of Field 1 were superior in terms of N accumulation. The cropping history only affected the P content in the grains of plants treated with SAF9 and SAF11, with Fields 1 and 2 being superior in the first treatment, and Field 5 in the second. 

Inoculation with plant-growth-promoting bacteria also affected the thousand-grain mass in Fields 1 and 5, where mean thousand-grain mass was high in plants inoculated with BiomaPhos, SAF9, and SAC36 (Table 4). The productivity was affected by the inoculation of rhizobacteria in Fields 2 and 5, with high mean values in plants inoculated with SAF9 and SAC36 in Field 2 and in those treated with SAF9 in Field 5. In contrast, inoculation affected the MBC in all evaluated fields in the 2019/2020 crop, and in Field 1, the highest MBC was observed in the soil where the crop plants were inoculated with SAC36. In Field 2, the soils of plants treated with BiomaPhos or SAC36 showed high concentration of MBC. In Field 5, however, the soil of plants treated with SAF9 presented the highest mean MBC, followed by the soil of plants treated with BiomaPhos and SAC36.

The thousand-grain mass of all plants differed in fields with different cropping histories, except for the control treatment, where values of thousand-grain mass were always higher for Fields 1 and 5 than those of Field 2 for all the tested bacteria. Similarly, the productivities of plants, including the controls, grown in Fields 1 and 5 were always greater than those grown in Field 2. The soil of plants grown in Fields 1 and 5 accumulated more MBC in plants treated with SAF11; however, in the control plants, or those treated with SAF9, the soil of Field 5 accumulated more MBC than soil in the other fields.

### 3.2. Results for the Summer Crop 2020/2021

Inoculation with the different rhizobacteria affected the plant height in only one of the evaluated fields in the 2020/2021 summer crop. In Field 3, plants inoculated with bacteria SAF9 and SAF11 were the tallest, followed by those treated with SAC36 and BiomaPhos (Table 5). In Field 3, APDM was high in plants treated with SAF11, SAC36, and BiomaPhos, and a similar behavior was observed in Field 4. Similarly, these treatments positively affected the RDM in plants grown in Fields 3 and 4. Regarding NN, however, plants treated with BiomaPhos in Field 3 and plants treated with BiomaPhos and SAF9 in Field 4 were the tallest. The NDM in these fields was positively affected by the treatment with BiomaPhos, but, in general, low vegetative measurements were observed in the non-inoculated plants (APDM in Field 4, NN in Field 4, and the NDM in Fields 3 and 4).

Plant height in the control and after inoculation with the bacteria SAF9, SAC36, and BiomaPhos differed in fields with different cultivation histories, with the highest mean values always observed in plants grown in Field 4. The cultivation history also affected APDM in all inoculation and control treatments, with the highest mean values found in plants of Field 4, mainly for the treatment without inoculation. The RDM followed a similar pattern. The mean NN values of plants in Field 4 were always higher, except for plants treated with SAF11, for which no effect was observed for cultivation field. Similarly, the observed mean values of NDM were always higher for plants grown in Field 4, except for those treated with the SAF11 bacterium.

The N levels in the aerial part were only affected by the inoculation of different rhizobacteri in Field 3 of the 2020/2021 crop year, with plants inoculated with SAC36 accumulating more N than those subjected to the other inoculation or non-inoculated treatments (Table 6). In contrast, the P content in the aerial part of the *G. max* plants was affected by the bacteria in both evaluated fields in the 2020/2021 crop year. In Field 3, plants treated with SAF11 showed the highest concentrations of P, followed by those treated with SAC36. In Field 4, plants treated with SAF9 and SAF11 showed the highest concentrations of P in the aerial parts. Grain N levels were only affected by inoculation treatments in Field 4, where reduced mean values of this nutrient were only observed in seeds from non-inoculated plants.

The P content in grains was affected by inoculation treatments in the two fields evaluated in the 2020/2021 crop year, with inoculation in Field 3 with SAF9 and SAF11, followed by BiomaPhos, increasing the P levels. In Field 4, however, lower mean values of P in grains were observed in plants treated with the commercial product BiomaPhos.

The N content in the aerial part was only affected by cultivation history in the control plants, where plants grown in Field 4 were superior, and in those treated with SAC36, where plants grown in Field 3 were superior. Similarly, the P content in the aerial part was affected by cultivation history, where plants treated with SAF9 and grown in Field 4 were superior to those grown in Field 3, and those treated with SAC36 and grown in Field 3 were superior to those cultivated in Field 4. The cultivation history only affected the grain N levels in plants with the control treatment, where N levels were higher in plants cultivated in Field 3, whereas grain P levels were only affected by the cultivation history in plants inoculated with the SAC36 bacterium, where plants cultivated in Field 4 accumulated greater P concentrations.

The different rhizobacteria also affected the mass of 1000 grains and productivity in the two fields evaluated in the 2020/2021 crop. In Field 3, plants treated with the bacteria SAC36, presented the highest mean values for the mass of 1000 grains, followed by those inoculated with BiomaPhos and SAF9 (Table 7).

In Field 4, however, the highest mean values for mass of 1000 grains were observed in plants treated with the commercial product BiomaPhos. Independent of the microorganism, inoculation treatments positively affected the rates of productivity; that is, the lowest averages were seen in the non-inoculated plants in both fields. However, the MBC was affected by inoculation treatments only in Field 3, following the same pattern as that of productivity. Thus, the soil obtained from inoculated plants was always richer in MBC than that from non-inoculated plants.

The mass of 1000 grains only differed among fields with different cultivation histories in plants inoculated with the strains SAF9 and BiomaPhos, with the highest mean values observed in plants grown in Field 4. A similar effect was observed for productivity, where plants inoculated with SAF9 and BiomaPhos and grown in Field 4 were more productive than those grown in Field 3.

In contrast, the soil from plants grown in Field 4 was more effective in concentrating MBC than the soil from plants grown in Field 3, with a significant difference observed for plants inoculated with SAF9, SAF11, and BiomaPhos.

### 3.3. Comparing Inoculation Methods: Soil vs. Seed

**Figure 3:**
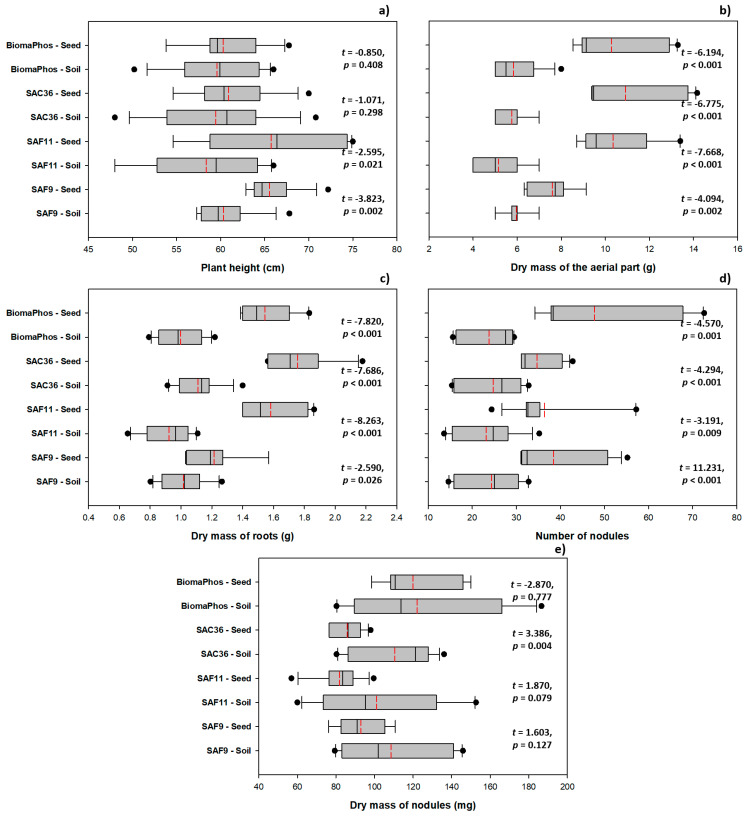
Effect of inoculation methods (via soil or seed treatment) of plant-growth-promoting rhizobacteria on aerial part height (**a**), aerial part dry mass (**b**), root dry mass (**c**), nodule number (**d**), and nodule dry mass (**e**) of *Glycine max* L. plants grown in summer 2019/2020 and 2020/2021, in five experimental soybean-growing fields in the interior of Goiás state, Brazil. SAF9, *Brevibacillus* sp.; SAF11, *Brevibacillus* sp.; SAC36, *Bacillus velezensis*; BiomaPhos, *Bacillus subtilis* CNPMS B2084 (BRM034840) and *Bacillus megaterium* CNPMS B119 (BRM033112).

The inoculation method only affected the height of G. max plants in treatments with the SAF9 and SAF11 strains, where seed inoculation was more effective than soil inoculation (Figure 3a). With SAF9, the mean plant heights were 60.35 and 65.53 cm with soil and seed inoculations, respectively, whereas with SAF11, the mean plant heights were 58.35 and 65.73 cm, respectively. Inoculation via seed was always superior to inoculation via soil for APDM, for all tested strains (Figure 3b). The mean APDM values for inoculation via soil and seed in plants treated with SAF9 were 5.96 and 7.56 g, respectively; 5.17 and 10.35 g, respectively, in those treated with SAF11; 5.75 and 10.91 g, respectively, in those inoculated with SAC36; and 5.83 and 10.29 g, respectively, in those inoculated with BiomaPhos. Similar effects were observed for RDM and NN (Figure 3c,d).

The observed mean RDM values were 1.02 g and 1.21 g for inoculation via soil and seed, respectively, in plants treated with SAF9; 0.92 g and 1.58 g, respectively, in those treated with SAF11; 1.11 g and 1.76 g, respectively, in those inoculated with SAC36; and 1.00 g and 1.54 g, respectively, in those inoculated with BiomaPhos. Mean NN values were 24.35 g and 38.45 g for soil and seed inoculation, respectively, in plants treated with SAF9; 23.13 g and 36.38 g, respectively, in those treated with SAF11; 24.74 g and 34.68 g, respectively, in those inoculated with SAC36; and 23.80 g and 47.71 g, respectively, in those inoculated with BiomaPhos. For NDM, however, the effect of inoculation method was only observed in plants inoculated with SAC36, with the highest mean values observed in plants inoculated via soil (0.11 mg) compared to plants inoculated via seed (0.09 mg) (Figure 3e).

**Figure 4:**
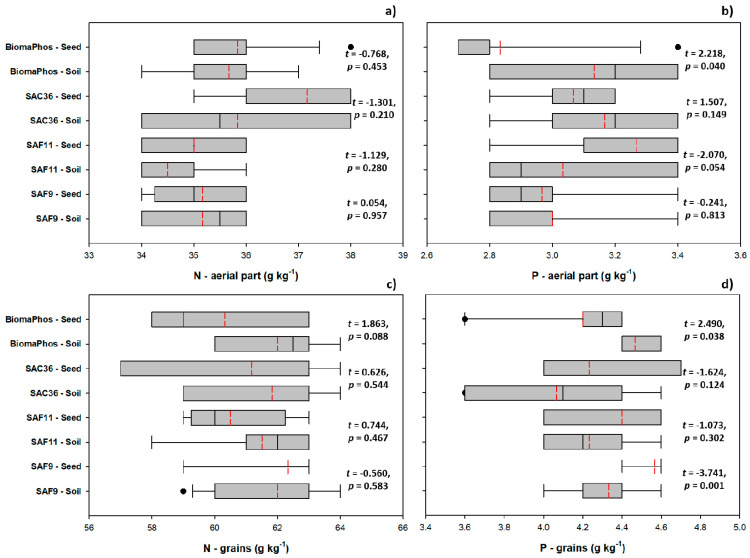
Effect of inoculation methods (via soil or seed treatment) of plant-growth-promoting rhizobacteria on the levels of N in the plant aerial part (**a**) and in the grains (**b**) and P in the aerial part (**c**) and in the grains (**d**) of *Glycine max* L. plants grown in summer 2019/2020 and 2020/2021, in five experimental fields of soybean cultivation in the interior of the state of Goiás, Brazil. SAF9, *Brevibacillus* sp.; SAF11, *Brevibacillus* sp.; SAC36, *Bacillus velezensis*; BiomaPhos, *Bacillus subtilis* CNPMS B2084 (BRM034840) and *Bacillus megaterium* CNPMS B119 (BRM033112).

Inoculation methods did not differentially affect the N content in the aerial part and grain of *G. max* (Figure 4a,c); however, the P content in the aerial part of plants inoculated with BiomaPhos showed higher mean values accumulated in plants inoculated via soil (0.31 g kg^−1^), than in plants inoculated via seed (0.28 g kg^−1^) (Figure 4b). The P content in the grains of plants treated with BiomaPhos was also affected by inoculation method, with the mean values being higher in plants inoculated via soil (0.45 and 0.42 g kg ^−1^ for soil- and seed-inoculated plants, respectively) (Figure 4d). The inoculation method also had an effect on grain P levels in plants treated with SAF9, with mean values of 0.43 and 0.46 g kg^−1^ for plants inoculated via soil and seed, respectively.

**Figure 5:**
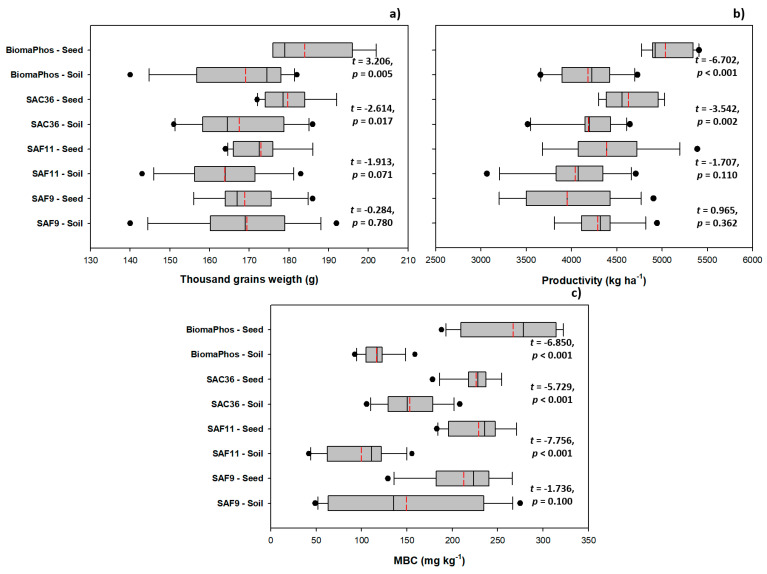
Effect of inoculation methods (via soil or seed treatment) of plant-growth-promoting rhizobacteria on thousand-grain mass (**a**), yield (**b**), and microbial biomass carbon (MBC) (**c**) sampled in the soil of *Glycine max* L. plants grown in summer 2019/2020 and 2020/2021, in five experimental soybean-growing fields in the interior of Goiás state, Brazil. SAF9, *Brevibacillus* sp.; SAF11, *Brevibacillus* sp.; SAC36, *Bacillus velezensis*; BiomaPhos, *Bacillus subtilis* CNPMS B2084 (BRM034840) and *Bacillus megaterium* CNPMS B119 (BRM033112).

The thousand-grain mass was affected by the inoculation method in plants treated with all tested strains, except SAF9. Mean values of 164.00 g and 173.00 g were observed for inoculation via soil and seed, respectively, in plants treated with SAF11; 167.46 g and 179.70 g, respectively, in those treated with SAC36; and 169.00 g and 184.00 g, respectively, in those inoculated with BiomaPhos, demonstrating the superiority of the inoculum-seed-treatment method (Figure 5a). Productivity, however, was only affected by the inoculation method in plants treated with SAC36 (4187.99 kg ha^−1^ and 4629.80 kg ha^−1^, seed and soil inoculation, respectively) and BiomaPhos (4181.12 kg ha^−1^ and 5035.47 kg ha^−1^, seed and soil inoculation, respectively); thus, higher mean values were observed in plants with inoculation via seed (Figure 5b). Mean MBC values of 99.73 mg kg^−1^ and 229.14 mg kg^−1^ were observed for soil and seed inoculation, respectively, in plants treated with SAF11; 153.49 mg kg^−1^ and 226.29 mg kg^−1^, respectively, in those treated with SAC36; and 116.55 mg kg^−1^ and 267.28 mg kg^−1^, respectively, in those inoculated with BiomaPhos, corroborating the hypothesis of the superiority of the inoculum-seed-treatment method compared to inoculation via soil (Figure 5c).

## 4. Discussion

The rhizobacteria obtained from *G. max* seedlings were more effective or demonstrated a similar effectiveness to the commercial product BiomaPhos for the majority of the vegetative, nutritional, and yield characteristics that were evaluated. In the fields tested in the 2019/2020 summer crop, the strains SAC36 of *Bacillus velezensis* and SAF9 of *Brevibacillus* sp. stood out as growth promoters, whereas, in the 2020/2021 season, SAF11 of *Brevibacillus* sp. SAC36 and BiomaPhos were notable.

The plant-growth-promoting action of *B. velezensis* has been confirmed in several studies; Adeniji et al. [42] highlighted the potential of *B. velezensis* for agricultural use, and Meng et al. [43], when testing the BAC03 strain, verified increased growth in nine selected types of plants, confirming its capacity to produce IAA and NH_3_, as well as showing ACC deaminase activity. Balderas-Ruíz et al. [44] and Myo et al. [45] indicated its potential for the biocontrol of phytopathogens. According to Rabbee et al. [46], *B. velezensis* possesses specific groups of genes related to the biosynthesis of secondary metabolites, which play significant roles in pathogen suppression and plant growth promotion. In addition, in *B. velezensis*, Chen et al. [47] observed clusters of genes responsible for antifungal metabolites (fengycin, surfactin, and bacillisin) and antibacterial metabolites (butyrosin, bacillaiene, diphifidin, macrolactin, surfactin, and bacillisin), in addition to various growth-promotion-related characteristics including phosphate solubilization, siderophores production, and root growth induction.

Bacteria of *Brevibacillus* are also known for their growth-promoting potential, which was attested to in several studies [48,49,50]. Wani et al. [51] and Ray et al. [52] discuss that this genus has high agroecological significance as potential plant-growth-promoting rhizobacteria, biocontrol agents against plant diseases, and for effective bioremediation to remove toxic heavy metals from soil, water, and atmosphere. In an in vitro study by Chakra et al. [53], the strain *Brevibacillus* sp. (SP-03), isolated from corn rhizosphere, showed characteristics of plant growth promotion, such as N_2_ fixation, IAA production, ammonia production, and siderophore production.

Similarly, the effectiveness of the BiomaPhos inoculant was confirmed for soybean and corn in several studies [54,55], and the present study corroborated its potential in ensuring good performances in soybean crops. However, overall, we observed a positive effect of inoculation on plant growth, where control plants showed low nodulation and low mean NDM in plants sampled from the 2019/2020 crop, and low mean APDM, nodulation, NDM, productivity, and MBC in those sampled from the 2020/2021 crop. This occurred despite these plants having received adequate planting fertilization, attesting to the importance of inoculating *G. max* plants with plant-growth-promoting rhizobacteria to ensure high levels of productivity [56,57] and decrease the costs of fertilization and chemical control [58,59].

The relationship between inoculation with strains of some rhizobacteria and the promotion of nodulation and nitrogen fixation in legumes is mediated through the bacterial production of flavonoid-type compounds or by stimulating the host legume to produce more flavonoid signaling molecules [60,61]. In contrast, auxin-producing rhizobacteria also stimulate nodule formation in several legume crops [56]. This may explain the nodulating effect of the rhizobacteria tested in this study, although many studies have reported a synergism between *Bradyrhizobium* (inoculant previously incorporated to the seeds used in the current experiment) and other rhizobacteria in nodulation and growth promotion in *G. max* [62,63,64,65]. For example, Masciarelli et al. [66], demonstrated the positive effect of *B. amyloliquefaciens* and *Bradyrhizobium japonicum* inoculation in increasing nodulation, which may explain the efficient NN and NDM values observed in this study, in plants treated with BiomaPhos.

In the 2019/2020 crop, Fields 1 and 2 stood out when the vegetative characters of growth and nutritional levels of plants were evaluated; however, Fields 1 and 5 were prominent with respect to the yield characteristics. Thus, although the mean values observed for various variables were dissimilar in fields with different cropping histories, there does not seem to be a direct relationship between longer periods of soybean cultivation as a monoculture and lower yield in the field. Yield, however, seems to be associated with the nutritional characteristics of the soil. Fields 1 and 2 concentrated the highest observed Ca, Mg, K, and P levels in the soil, and the highest cation exchange capacity, which is accompanied by the low leaching of cations. These characteristics may explain the development and accumulation of nutrients in plants grown in these fields. However, in Field 2, unlike in Field 1, the increased accumulation of N and P in the aerial part and grains observed in the plants treated with some of the rhizobacteria did not result in increased productivity. In Field 5, the cultivation history, namely, the first year of soybean planting, explains the high concentrations of MBC observed even in the soil where the control plants were grown. This MBC represents the remaining microbial biomass from previous plantations of the forage crop *Brachiaria ruziziensis*. According to Singh and Gupta [67], soil microbial biomass plays an important role in the nutrient dynamics and productivity of ecosystems. The high levels of available carbon sustained high production in Field 5. However, because it was the first year of soybean cultivation, this field likely did not have an established population of diazotrophs, which may explain the low root nodulation observed in plants grown in this Field.

In the 2020/2021 crop, compared to Field 3, plants grown in Field 4, exhibited excellent vegetative characteristics of development, mass of 1000 grains and productivity. Field 3 was the most dissimilar among the fields, with soils containing low amounts of Ca, Mg, K, and P, high levels of Fe, and a very acidic pH. Acidic pH interferes with the uptake of nutrients, with absorption of Fe, Cu, Mn, Zn, Co, and Ni being increased under this condition, and because pH needs to be close to neutral for the best uptake of essential nutrients such as N, P, S, K, Ca, and Mg [68]. This may explain the lower productivity observed in Field 3 compared to Field 4, which was the richest in OM among all the fields. Recently, Oldfield et al. [69] showed a direct relationship between soil OM concentration and crop yield, inferring that increases in yield stabilize at ~2% soil organic carbon. The problem is that approximately two-thirds of the world’s cultivated maize and wheat lands currently have soil organic carbon levels below 2%.

The MBC was positively influenced by inoculation of SAC36 in Fields 1 and 2; by SAC36, SAF9, and BiomaPhos in Field 5; and by all inoculation treatments in Field 3. High MBC values indicate that nutrients are temporarily immobilized, which results in lower losses of these nutrients in the soil–plant system [70]. The results of this study show that the inoculation of *G. max* with the tested rhizobacteria favors the MBC owing to nutrient cycling [71], mainly in fields with low levels of P (Field 3).

A strong effect of the inoculation method was observed on APDM, RDM, NN, and MBC, with the plants inoculated via seed showing higher means for these variables than plants inoculated via soil. This is because the survivability of the strains can be affected when bacterial inoculants are sprayed on the soil, as they become more susceptible to biotic and abiotic stresses [72]. In the soil, inoculants also compete with the resident microbiota, and microorganisms in the receiving environment are highly diverse and better adapted than an introduced microorganism [73]. O’Callaghan [35] explains that the application of beneficial microorganisms to seed is an efficient mechanism for allocating microbial inocula to soil, and these microorganisms will be well-positioned to colonize seedling roots and protect against soil-borne diseases and pests.

Although the long history of inoculating legume seed with *Rhizobium* is accompanied by a clear laboratory demonstration of the ability of a wide range of other beneficial microorganisms to improve crop performance, very few microbial inoculants are commercially available [35]. As the isolates tested here were sometimes superior or similar to the action of the phosphate-solubilizing inoculant BiomaPhos on the growth and productivity of *G. max*, there is the possibility that these strains, especially SAC36, will eventually be incorporated into the biostimulants market, contributing to the technological development of agriculture and the consolidation of eco-friendly agricultural technologies.

## 5. Conclusions

This study confirmed the hypothesis that phosphate-solubilizing rhizobacteria previously isolated from *G. max* seedlings can be effective in promoting the growth of plants of this species in crops cultivated in soils with different oilseed cultivation histories. Thus, in the 2019/2020 summer crop, the strains SAC36 of *Bacillus velezensis* and SAF9 of *Brevibacillus* sp. were notable as growth promoters, whereas, in the 2020/2021 season, SAF11 of *Brevibacillus* sp., SAC36, and BiomaPhos were prominent. The tested rhizobacteria were sometimes more effective than, and sometimes as effective as, the commercial product BiomaPhos. In contrast, no direct relationship was observed between a longer periods of soybean cultivation as a monoculture and lower yield in the field, whereas yield was possibly associated with the nutritional conditions of the soils (as observed in the plants grown in the nutritionally more favorable Fields 1 and 2 in the 2019/2020 crop and in Field 4, which was rich in OM, in the 2020/2021 crop). The hypothesis of the superiority of the inoculation method via seeds was confirmed, as it ensured higher nodulation and a higher NDM than soil inoculation. This study confirmed the growth-promoting potential of new phosphate-solubilizing strains, which may eventually be incorporated by the biostimulants market to freely compete with BiomaPhos.

## Figures and Tables

**Table 1:** Multifunctional characterization of SAF9, SAF11, and SAC36 rhizobacteria collected from *Glycine max* L. seedlings grown in soil sampled from an agricultural field with a 30-year history of soybean cultivation.

Functional Traits		*Brevibacillus* sp.	*Brevibacillus* sp.	*Bacillus velezensis*
		(SAF9)	(SAF11)	(SAC36)
Solubilization of CaHPO_4_	(mg L^−1^)	12.2	10.5	10.8
Solubilization of FePO_4_	(mg L^−1^)	8.4	7.4	6.6
IAA synthesis	(µg mL^−1^)	11.4	16.9	13.7
GA_3_ synthesis	(µg mL^−1^)	211.2	385.6	226.6
*Fusarium* sp. *Sclerotinia sclerotiorum*	(R.I.%)	33.6	44.2	37.2
	(R.I.%)	51.8	38.2	37.2
Production of siderophores		−	−	+

CaHPO_4_, calcium phosphate; FePO_4_, iron phosphate; IAA, indole acetic acid; GA_3_, gibberellic acid; R.I.%, percentage of relative inhibition of mycelial growth; (+), positive for siderophore production; (−), negative for siderophore production.

**Table 2:** Effect of inoculation with plant-growth-promoting rhizobacteria on aerial part height (APH), aerial part dry mass (APDM), root dry mass (RDM), number of nodules (NN), and nodule dry mass (NDM) on *Glycine max* L. plants grown in summer 2019/2020, in three experimental fields of soybean cultivation, in the interior of the state of Goiás, Brazil, with different soybean cultivation time histories: Field 1, soybean production for 30 years; Field 2, soybean production for 15 years; and Field 5, first year of soybean cultivation.

	**Field 1**	**Field 2**	**Field 5**		**Field 1**	**Field 2**	**Field 5**		**Field 1**	**Field 2**	**Field 5**	
**Treatments**	**APH (cm)**			**CV (%)**	**APDM (g)**			**CV (%)**	**RDM (g)**			**CV (%)**
**Control**	54.48 ± 0.52 NSns	59.50 ± 2.11 NSns	59.21 ± 1.62 NSns	7.03	5.25 ± 0.12 NSns	5. 50 ± 0.43 NSns	4.13 ± 0.36 Bns	19.75	0. 87 ± 0.03 NSns	0.87 ± 0.05 NSns	0.92 ± 0.03 Bns	10.77
**SAF9**	58.50 ± 0.90 NSns	60.85 ± 2.01 NSns	61.69 ns ± 0.47 NSns	4.62	5.50 ± 0.25 NSns	6.13 ± 0.25 NSns	6.25 ± 0.21 Ans	8.59	0.91 ± 0.03 NSns	1.08 ± 0.05 NSns	1.05 ± 0.07 ABns	15.19
**SAF11**	52.85 ± 1.60 NSns	58.85 ± 3.56 NSns	63.35 ± 0.60 NSns	7.09	4.75 ± 0.64 NSns	5.50 ± 0.55 NSns	5.25 ± 0.21 ABns	15.93	0.81 ± 0.07 NSns	0.97 ± 0.05 NSns	0.99 ± 0.04 ABns	11.79
**SAC36**	52.70 ± 1.42 NSns	65.95 ± 1.41 NSns	59.70 ± 1.50 NSns	5.05	5.50 ± 0.25 NSns	5.50 ± 0.43 NSns	6.25 ± 0.21 Ans	11.79	1.02 ± 0.04 NSns	1.08 ± 0.04 NSns	1.23 ± 0.04 Ans	9.40
**BiomaPhos**	54.30 ± 20 NSns	66.45 ± 0.34 NSns	59.65 ± 0.38 NSns	1.42	5.50 ± 0.43 NSns	6.50 ± 0.43 NSns	5.50 ± 0.43 ABns	17.14	0.84 ± 0.01 NSns	1.08 ± 0.04 NSns	1.07 ± 0.05 ABns	11.31
**CV (%)**	5.06	8.05	4.04		16.88	17.20	12.86		11.92	11.54	11.99	
			**Field 1**	**Field 2**	**Field 5**		**Field 1**	**Field 2**	**Field 5**			
		**Treatments**	**NN**			**CV (%)**	**NDM (mg)**			**CV (%)**		
		**Control**	14.40 ± 0.68 Cb	19.29 ± 0.55 Ba	5.30 ± 0.15 Bc	7.93	32.50 ± 1.14 Cb	70.00 ± 0.60 Ba	33.00 ± 0.90 Cb	20.14		
		**SAF9**	31.33 ± 0.64 Aa	2.25 ± 1.26 ABa	15.47 ± 0.39 Ab	9.66	134.52 ± 2.27 ABa	111.50 ± 0.80 Ab	82.00 ± 0.10 ABc	13.49		
		**SAF11**	25.65 ± 1.29 Ba	28.90 ± 1.93 Aa	14.85± 0.38 Ab	13.71	110.95 ± 2.85 Ba	120.00 ± 0.92 Aa	71.00 ± 0.40 Bb	16.42		
		**SAC36**	27.85 ± 0.80 ABa	30.60 ± 1.18 Aa	15.76 ± 0.18 Ab	7.85	122.50 ± 2.31 ABa	120.00 ± 0.14 Aa	85.00 ± 0.22 Ab	4.71		
		**BiomaPhos**	27.94 ± 0.20 ABa	27.43 ± 1.73 Aa	16.03 ± 0.16 Ab	11.81	169.40 ± 0.60 Aa	111.00 ± 0.49 Ab	85.00 ± 0.25 Ac	8.65		
		**CV (%)**	7.31	12.37	4.82		19.57	14.85	8.01			

Control, no inoculation; SAF9, *Brevibacillus* sp.; SAF11, *Brevibacillus* sp.; SAC36, *Bacillus velezensis*; BiomaPhos, *Bacillus subtilis* CNPMS B2084 (BRM034840) and *Bacillus megaterium* CNPMS B119 (BRM033112). NS = not significant; CV = coefficient of variation. Capital letters compare isolates in the column and lowercase letters compare fields in the rows. Mean followed by different letters, in the column or row, differed by the Tukey test (5%).

**Table 3:** Effect of inoculation with plant-growth-promoting rhizobacteria on nitrogen (N) and phosphorus (P) contents in the aerial part and grains of *Glycine max* L. plants grown in summer 2019/2020, in three experimental fields of soybean cultivation, in the interior of the state of Goiás, Brazil, with different soybean cultivation histories: Field 1, soybean production for 30 years; Field 2, soybean production for 15 years; and Field 5, first year of soybean cultivation.

	**Field 1**	**Field 2**	**Field 5**		**Field 1**	**Field 2**	**Field 5**	
**Treatments**	**N Aerial Part (g kg^−1^)**			**CV (%)**	**P Aerial Part (g kg^−1^)**			**CV (%)**
**Control**	35.50 ± 0.25 NSa	35.00 ± 0.75 Ba	33.50 ± 0.75 NSb	4.09	3.10 ± 0.15 Bns	3.00 ± 0.10 NSns	3.00 ± 0.00 NSns	7.75
**SAF9**	35.50 ± 0.50 NSns	35.50 ± 0.25 ABns	35.00 ± 0.50 NSns	2.84	3.20 ± 0.06 Aa	2.90 ± 0.05 NSb	2.90 ± 0.05 NSb	5.44
**SAF11**	34.50 ± 0.25 NSns	34.00 ± 0.00 Cns	35.00 ± 0.50 NSns	2.16	2.90 ± 0.05 Bns	3.10 ± 0.15 NSns	3.10 ± 0.15 NSns	9.59
**SAC36**	36.00 ± 0.10 NSa	37.00 ± 0.50 Aa	34.50 ± 0.25 NSb	5.76	3.20 ± 0.10 Ans	3.10 ± 0.15 NSns	3.20 ± 0.10 NSns	8.68
**BiomaPhos**	36.00 ± 0.25 NSns	35.00 ± 0.50 Bns	35.50 ± 0.25 NSns	2.29	3.10 ± 0.15 Bab	3.40 ± 0.00 NSa	2.90 ± 0.05 NSb	6.73
**CV (%)**	3.71	2.93	3.37		8.97	7.71	7.35	
	**Field 1**	**Field 2**	**Field 5**		**Field 1**	**Field 2**	**Field 5**	
**Treatments**	**N grain (g kg^−1^)**			**CV (%)**	**P grain (g kg^−1^)**			**CV (%)**
**Control**	61.00 ± 1.00 Ba	60.50 ± 0.25 Ba	57.00 ± 0.25 Bb	2.75	4.00 ± 0.05 NSns	4.10 ± 0.00 Bns	4.30 ± 0.15 NSns	5.76
**SAF9**	60.50 ± 0.40 Bb	63.50 ± 0.25 Aa	62.00 ± 0.10 Aab	2.15	4.40 ± 0.00 NSa	4.50 ± 0.05 Aa	4.10 ± 0.05 NSb	2.18
**SAF11**	62.00 ± 0.30 Ba	62.00 ± 0.20 ABa	60.50 ± 0.12 ABb	3.11	4.00 ± 0.00 NSb	4.20 ± 0.10 ABb	4.50 ± 0.05 NSa	3.52
**SAC36**	64.00 ± 0.20 Aa	61.00 ± 0.10 Bb	61.00 ± 0.10 ABb	3.10	4.00 ± 0.02 NSns	3.90 ± 0.15 Cns	4.30 ± 0.15 NSns	9.56
**BiomaPhos**	61.00 ± 0.20 Bb	63.50 ± 0.20 Aa	61.50 ± 0.50 ABab	2.01	4.50 ± 0.00 NSns	4.40 ± 0.00 ABns	4.50 ± 0.05 NSns	2.11
**CV (%)**	2.35	2.18	3.68		5.40	5.78	5.32	

Control, no inoculation; SAF9, *Brevibacillus* sp.; SAF11, *Brevibacillus* sp.; SAC36, *Bacillus velezensis*; BiomaPhos, *Bacillus subtilis* CNPMS B2084 (BRM034840) and *Bacillus megaterium* CNPMS B119 (BRM033112). NS = not significant; CV = coefficient of variation. Capital letters compare isolates in the column and lowercase letters compare fields in the rows. Mean followed by different letters, in the column or row, differed by the Tukey test (5%).

**Table 4:** Effect of inoculation with plant=growth-promoting rhizobacteria on thousand-grain mass, yield, and microbial biomass carbon (MBC) sampled in the soil of *Glycine max* L. plants grown in summer 2019/2020, in three experimental fields of soybean cultivation in the interior of the state of Goiás, Brazil, with different histories of soybean cultivation: Field 1, soybean production for 30 years; Field 2, soybean production for 15 years; and Field 5, first year of soybean cultivation.

	**Field 1**	**Field 2**	**Field 5**		**Field 1**	**Field 2**	**Field 5**	
**Treatments**	**Mass of One Thousand Grains (g)**			**CV (%)**	**Productivity (kg ha^−1^)**			**CV (%)**
**Control**	164 ± 1.14 Bns	155 ± 8.28 NSns	174 ± 2.13 Bns	7.61	4133.93 ± 38 66 NSa	3440.63 ± 30.80 Cb	4242.50 ± 103.30 Ba	6.68
**SAF9**	169 ± 2.27 ABa	155 ± 4.86 NSb	180 ± 0.8 Aa	4.78	4337.50 ± 68.80 NSa	4007.29 ± 99.71 Ab	4526.25 ± 28.08 Aa	6.33
**SAF11**	164 ± 2.85 Ba	153 ± 3.20 NSb	174 ± 2.10 Ba	4.35	4133.93 ± 109.13 NSa	3645.83 ± 99.21 Bb	4348.75 ± 48.30 Ba	9.38
**SAC36**	165 ± 2.31 ABa	156 ± 2.24 NSb	178 ± 0.50 ABa	2.41	4206.25 ± 27.58 NSa	4070.83 ± 33.34 Ab	4286.87 ± 24.40 Ba	10.89
**BiomaPhos**	177 ± 0.61 Aa	153 ± 3.73 NSb	180 ± 0.80 Aa	3.06	4436.07 ± 141.61 NSa	3779.17 ± 58.35 Bb	4328.13 ± 81.73 Ba	4.16
**CV (%)**	2.77	7.37	3.24		4.80	8.08	7.74	
			**Field 1**	**Field 2**	**Field 5**			
		**Treatments**	**BC (mg Kg^−1^)**			**CV (%)**		
		**Control**	103.96 ±4.61 Bb	71.39 ± 6.76 Bc	162.41 ± 21.86 Ba	16.73		
		**SAF9**	142.87 ± 15.02 Bb	59.43 ± 4.46 Bc	247.06 ± 20.04 Aa	15.93		
		**SAF11**	119.89 ±15.15 Ba	56.66 ± 9.26 Bb	122.65 ± 10.54 Ba	23.04		
		**SAC36**	186.25 ± 22.53 Aa	147.98 ± 4.53 Aab	123.26 ± 19.78 ABb	12.48		
		**BiomaPhos**	110.66 ± 5.33 Bns	114.63 ± 4.65 Ans	124.65 ± 27.18 ABns	18.07		
		**CV (%)**	10.84	12.40	9.47			

Control, no inoculation; SAF9, *Brevibacillus* sp.; SAF11, *Brevibacillus* sp.; SAC36, *Bacillus velezensis*; BiomaPhos, *Bacillus subtilis* CNPMS B2084 (BRM034840) and *Bacillus megaterium* CNPMS B119 (BRM033112). NS = not significant; CV = coefficient of variation. Capital letters compare isolates in the column and lowercase letters compare fields in the rows. Mean followed by different letters in the column differed by the Tukey test (5%).

**Table 5:** Effect of inoculation with plant-growth-promoting rhizobacteria on aerial part height (APH), aerial part dry mass (APDM), root dry mass (RDM), number of nodules (NN), and nodule dry mass (NDM) of *Glycine max* L. plants grown in the summer 2020/2021, in two experimental fields of soybean cultivation, in the interior of the state of Goiás, Brazil, with different histories of soybean cultivation: Field 3, soybean production for 10 years and Field 4, second year of soybean cultivation.

	**Field 3**	**Field 4**		**Field 3**	**Field 4**		**Field 3**	**Field 4**	
**Treatments**	**APH (cm)**		**Test *t***	**APDM (g)**		**Test *t***	**RDM (g)**		**Test *t***
**Control**	52.34 ± 1.99 B	64.10 ± 1.14 NS	*t* = −4.42, *p* = 0.007	7.16 ± 0.30 B	6.19 ± 0.35 C	*t* = 34.42, *p* < 0.001	1.05 ± 0.03 B	1.15 ± 0.04 B	*t* = 1.43, *p* = 0.205
**SAF9**	64.01 ± 0.45 A	68.33 ± 1.14 N	*t* = −3.02, *p* = 0.039	7.11 ± 0.37 B	8.25 ± 0.26 B	*t* = −2.15, *p* = 0.079	1.11 ± 0.04 B	1.36 ± 0.06 B	*t* = −2.94, *p* = 0.030
**SAF11**	63.60 ± 3.81 A	70.00 ± 2.21 NS	*t* = −1.26, *p* = 0.266	9.24 ± 0.18 A	12.57 ± 0.41 A	*t* = 6.34, *p* = 0.002	1. 46 ± 0.04 A	1.83 ± 0.01 A	*t* = −6.77, *p* = 0.002
**SAC36**	58.40 ± 1.34 AB	65.95 ± 1.30 NS	*t* = −3.48, *p* = 0.013	9.43 ± 0.02 A	13.88 ± 0.09 A	*t* = 37.74, *p* < 0.001	1. 64 ± 0.04 A	2.00 ± 0.07 A	*t* = −3.66, *p* = 0.014
**BiomaPhos**	58.01 ± 1.25 AB	64.82 ± 1.40 NS	*t* = −3.12, *p* = 0.020	8.94 ± 0.13 A	12.98 ± 0.17 A	*t* = 16.36, *p* < 0.001	1. 44 ± 0.02 A	1.75 ± 0.04 A	*t* = −5.09, *p* = 0.005
**CV (%)**	8.20	6.99		6.60	6.12		6.57	7.47	
		**Field 3**	**Field 4**		**Field 3**	**Field 4**			
	**Treatments**	**NN**		**Test *t***	**NDM (mg)**		**Test *t***		
	**Control**	13.23 ± 0.52 B	22.16 ± 0.28C	*t* = −13.01, *p* < 0.001	30.00 ± 2.50 C	43.00 ± 0.80 D	*t* = 21.76, *p* < 0.001		
	**SAF9**	31.76 ± 0.38 B	55.15 ± 2.21 AB	*t* = −7.38, *p* = 0.004	87.00 ± 5.50 B	102.00 ± 2.46 B	*t* = 71.13, *p* < 0.001		
	**SAF11**	32.79 ± 0.30 B	43.54 ± 7.02 B	*t* = −1.32, *p* = 0.276	83.00 ± 2.34 B	79.00 ± 8.60 C	*t* = 3.35, *p* = 0.043		
	**SAC36**	31.55 ± 0.23 B	40.95 ± 0.54 B	*t* = −13.73, *p* < 0.001	83.00 ± 3.35 B	92.00 ± 1.99 BC	*t* = 115.51, *p* < 0.001		
	**BiomaPhos**	37.00 ± 0.88 A	68.00 ± 2.50 A	*t* = −10.09, *p* < 0.001	107.00 ± 2.53 A	146.00 ± 3.54 A	*t* = 36.24, *p* < 0.001		
	**CV (%)**	4.10	17.86		10.29	11.11			

Control, no inoculation; SAF9, *Brevibacillus* sp.; SAF11, *Brevibacillus* sp.; SAC36, *Bacillus velezensis*; BiomaPhos, *Bacillus subtilis* CNPMS B2084 (BRM034840) and *Bacillus megaterium* CNPMS B119 (BRM033112). NS = not significant; CV = coefficient of variation. Capital letters compare isolates in the column and lowercase letters compare fields in the rows. Mean followed by different letters in the column differed by the Tukey test (5%).

**Table 6:** Effect of inoculation with plant-growth-promoting rhizobacteria on N and P levels in the aerial part and grains of *Glycine max* L. plants grown in the summer 2020/2021, in two experimental fields of soybean cultivation, in the interior of the state of Goiás, Brazil, with different histories of soybean cultivation: Field 3, soybean production for 10 years and Field 4, second year of soybean cultivation.

	**Field 3**	**Field 4**		**Field 3**	**Field 4**	
**Treatments**	**N Aerial Part (g kg^−1^)**		**Test *t***	**P Aerial Part (g kg^−1^)**		**Test *t***
**Control**	34.00 ± 0.00 C	35.30 ± 0.21 NS	*t* = −5.00, *p* = 0.015	3.00 ± 0.01 BC	2.90 ± 0.05 B	*t* = 1.73, *p* = 0.187
**SAF9**	35.50 ± 0.25 B	35.00 ± 0.50 NS	*t* = 0.77, *p* = 0.478	2.90 ± 0.05 BC	3.30 ± 0.12 A	*t* = 2.17, *p* = 0.097
**SAF11**	35.00 ± 0.50 BC	35.00 ± 0.50 NS	*t* = 0.00, *p* = 0.875	3.30 ± 0.12 A	3.30 ± 0.08 A	*t* = 0.27, *p* = 0.792
**SAC36**	38.00 ± 0.00 A	35.50 ± 0.25 NS	*t* = 8.66, *p* = 0.003	3.20 ± 0.04 AB	2.90 ± 0.05 B	*t* = −3.27, *p* = 0.017
**BiomaPhos**	35.50 ± 0.25 B	36.00 ± 0.43 NS	*t* = −1.73, *p* = 0.146	2.80 ± 0.02 C	3.00 ± 0.12 B	*t* = 1.73, *p* = 0.174
**CV (%)**	1.78	2.60		5.07	7.09	
	**Field 3**	**Field 4**		**Field 3**	**Field 4**	
**Treatments**	**N grain (g kg^−1^)**		**Test *t***	**P grain (g kg^−1^)**		**Test *t***
**Control**	58.00 ± 0.01 NS	55.80 ± 0.64 B	*t* = −3.00, *p* = 0.057	4.00 ± 0.01 B	4.40 ± 0.01 AB	*t* = 0.10, *p* = 0.920
**SAF9**	62.000 ± 0.08 NS	62.00 ± 0.86 A	*t* = 0.00, *p* = 0.848	4.60 ± 0.04 A	4.60 ± 0.04 AB	*t* = 0.00, *p* = 0.965
**SAF11**	59.80 ± 0.21 NS	62.00 ± 0.86 A	*t* = 2.18, *p* = 0.107	4.50 ± 0.12 A	4.30 ± 0.15 AB	*t* = −0.65, *p* = 0.537
**SAC36**	61.50 ± 1.20 NS	60.50 ± 1.75 AB	*t* = −0.39, *p* = 0.706	4.00 ± 0.01 B	4.70 ± 0.01 A	*t* = 27.00, *p* < 0.001
**BiomaPhos**	60.50 ± 1.25 NS	60.00 ± 0.86 AB	*t* = −0.28, *p* = 0.786	4.30 ± 0.05 AB	4.00 ± 0.02 B	*t* = −1.26, *p* = 0.287
**CV (%)**	3.44	4.12		3.52	5.57	

Control, no inoculation; SAF9, *Brevibacillus* sp.; SAF11, *Brevibacillus* sp.; SAC36, *Bacillus velezensis*; BiomaPhos, *Bacillus subtilis* CNPMS B2084 (BRM034840) and *Bacillus megaterium* CNPMS B119 (BRM033112). NS = not significant; CV = coefficient of variation. Capital letters compare isolates in the column and lowercase letters compare fields in the rows. Mean followed by different letters in the column differed by the Tukey test (5%).

**Table 7:** Effect of inoculation with plant-growth-promoting rhizobacteria on thousand-grain mass, yield, and microbial biomass carbon (MBC) sampled in the soil of *Glycine max* L. plants grown in summer 2020/2021, in two experimental fields of soybean cultivation in the interior of the state of Goiás, Brazil, with different histories of soybean cultivation: Field 3, soybean production for 10 years and Field 4, second year of soybean cultivation.

	**Field 3**	**Field 4**		**Field 3**	**Field 4**	
**Treatments**	**Mass of One Thousand Grains (g)**		**Test *t***	**Productivity (kg ha^−1^)**		**Test *t***
**Control**	167 ± 4.09 AB	172 ± 1.73 B	*t =* −0. 97, *p* = 0.384	3138.19 ± 100.36 B	3721.18 ± 205.77 B	*t =* −2. 20, *p* = 0.086
**SAF9**	164 ± 2.28 B	177 ± 3.78 B	*t* = −2.65, *p* = 0.043	3652.08 ± 168.81 AB	4561.46 ± 99.55 A	*t* = −4.01, *p* = 0.010
**SAF11**	175 ± 3.54 AB	167 ± 1.29 B	*t* = 1.89, *p* = 0.135	4299.66 ± 205.85 AB	4561.11 ± 265.70 A	*t* = −0.67, *p* = 0.527
**SAC36**	182 ± 3.32 A	174 ± 1.24 B	*t* = 1.86, *p* = 0.139	4522.92 ± 145.93 A	4843.58 ± 49.99 A	*t* = −1.80, *p* = 0.152
**BiomaPhos**	177 ± 0.82 AB	197 ± 3.77 A	*t =* −4.36, *p* = 0.018	4879.41 ± 31.53 A	5347.40 ± 48.20 A	*t =* −7. 03, *p* < 0.001
**CV (%)**	4.10	3.43		8.10	8.01	
			**Field 3**	**Field 4**		
		**Treatments**	**MBC (mg Kg^−1^)**		**Test *t***	
		**Control**	155.01 ± 9.96 B	154.20 ± 13.18 NS	*t =* −0.03, *p* = 0.970	
		**SAF9**	238.58 ± 13.54 A	154.48 ± 12.59 NS	*t* = −3.71, *p* = 0.020	
		**SAF11**	247.62 ± 9.93 A	192.18 ± 5.97 NS	*t* = −3.90, *p* = 0.025	
		**SAC36**	237.10 ± 7.72 A	204.67 ± 10.77 NS	*t* = −1.99, *p* = 0.123	
		**BiomaPhos**	298.43 ± 16.47 A	204.40 ± 7.84 NS	*t =* −4. 18, *p* = 0.027	
		**CV (%)**	11.41	13.40		

Control, no inoculation; SAF9, *Brevibacillus* sp.; SAF11, *Brevibacillus* sp.; SAC36, *Bacillus velezensis*; BiomaPhos, *Bacillus subtilis* CNPMS B2084 (BRM034840) and *Bacillus megaterium* CNPMS B119 (BRM033112). NS = not significant; CV = coefficient of variation. Capital letters compare isolates in the column and lowercase letters compare fields in the rows. Mean followed by different letters in the column differed by the Tukey test (5%).

## Data Availability

All the data relevant to this manuscript are available on request from the corresponding author.
